# Congenital lobar emphysema

**DOI:** 10.1590/0100-3984.2017.0138

**Published:** 2019

**Authors:** Tiago Kojun Tibana, Denise Maria Rissato Camilo, Thiago Franchi Nunes, Edson Marchiori

**Affiliations:** 1 Universidade Federal de Mato Grosso do Sul (UFMS), Campo Grande, MS, Brazil.; 2 Universidade Federal do Rio de Janeiro (UFRJ), Rio de Janeiro, RJ, Brazil.

**Figure 1:**
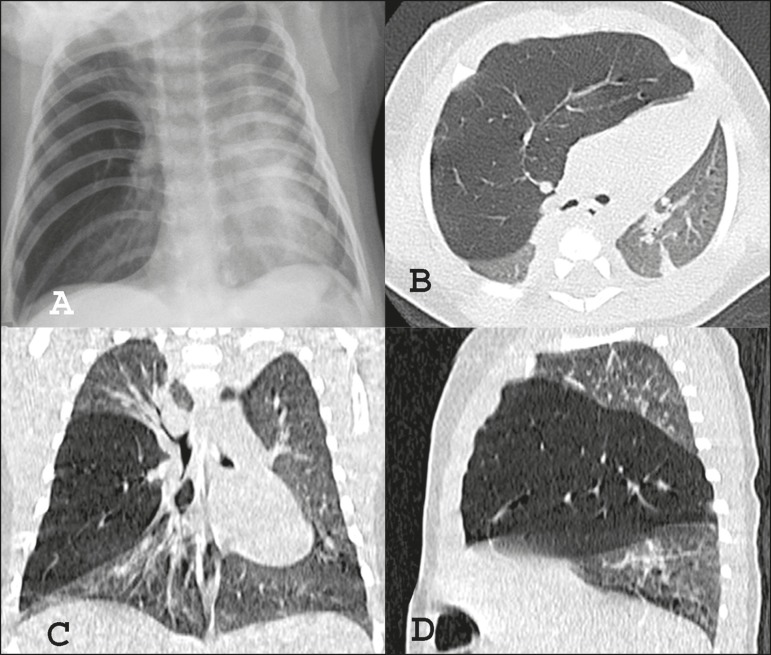
Chest X-ray in anteroposterior view (**A**) showing hyperlucency of the right hemithorax with a mediastinal shift to the left. Noncontrast chest CT, in axial, coronal, and sagittal slices (**B, C**, and **D**, respectively), showing hyperinflation of the middle lobe and expansion of the mediastinal structures.

Dear Editor,

A 41-day-old male infant was born by cesarean section, without complications, at 38 weeks of gestation. The results of the prenatal examinations had been normal, and postnatal nutrition was exclusively from breastfeeding. He was referred to our facility with a history of progressive respiratory distress, which had started on postnatal day 7 and had worsened three days prior to the consultation. He was afebrile. The parents reported having previously sought treatment more than once and having received a prescription for nebulization, which resulted in partial improvement of the condition. The initial physical examination revealed subcostal retraction, diminished breath sounds on the right side and diffuse wheezing on the left. The respiratory rate was 72 breaths/min, and the oxygen saturation on room air was 96%. A chest X-ray (Figure 1A) showed right-sided hyperlucency, with a mediastinal shift to the left. Computed tomography (CT) revealed hyperinflation of the middle lobe parenchyma, the expansion of which was displacing the mediastinum to the left (Figures 1B, 1C, and 1D). The patient was treated with a nebulized bronchodilator and oxygen therapy, which resulted in clinical improvement and stabilization of the condition. At five days after admission, he was asymptomatic and was discharged to outpatient follow-up.

Congenital diseases have been the subject of recent publications in the area of radiology^(^1^-^4^)^. Congenital lobar emphysema (CLE) is a rare pulmonary malformation whose main cause is probably developmental anomalies of the bronchial cartilage. Less common causes include extrinsic airway compression, usually caused by idiopathic bronchial stenosis, mucus plugging, or vascular malformations. However, in approximately half of all cases, the cause goes undetermined^(^5^-^10^)^.

CLE is characterized by progressive lobar hyperinflation, caused by air trapping in a collapsed airway, resulting in distension of the lobe and a mass effect that compresses the other lobes and shifts the mediastinum^(^6^,^^7^^)^. There is no alveolar destruction^(^11^)^. CLE involves the left upper lobe in 42.2% of cases, the right middle lobe in 35.3%, the right upper lobe in 20.7%, and the lower lobes in less than 1.0%^(^11^,^^12^^)^. Its clinical presentation ranges from mild respiratory dysfunction to acute respiratory failure. Most patients are diagnosed within the first month of life, showing a moderate degree of respiratory dysfunction in the immediate postnatal period, and present symptoms before reaching six months of age, with progressive worsening due to increased pulmonary hyperinflation. Some patients remain asymptomatic for years^(^5^,^^10^^,^^11^^)^.

A diagnosis of CLE is generally suspected in a child with respiratory failure in whom a chest X-ray reveals hyperinflation of a lung lobe, with or without contralateral pulmonary herniation, and a contralateral mediastinal shift^(^7^,^^10^^)^. CT is an excellent imaging modality for excluding diagnoses of a subjacent hilar mass and alterations in the bronchial lumen. In addition, it can accurately delineate and localize the lesion, which is particularly useful for preoperative evaluation. CT usually shows hyperinflation of a lung lobe and attenuation of the bronchovascular bundle, which runs along the periphery of the expanded alveoli^(^10^,^^11^^)^. The differential diagnosis includes pneumatocele, pneumothorax, pulmonary atelectasis, and pulmonary hypoplasia.

CLE is generally considered an indication for surgery, lobectomy being the procedure of choice in symptomatic patients^(^5^,^^10^^)^. For patients who exhibit mild respiratory distress, conservative treatment is an option^(^3^)^.
